# Supplementary material for: Potent Anticancer Activity of CXCR4-Targeted Nanostructured Toxins in Aggressive Endometrial Cancer Models
Source: Cancers (Basel). 2022 Dec 23;15(1):85. doi: 10.3390/cancers15010085 (PMC9818013; doi:10.3390/cancers15010085)
Supplement: Supplementary file 1 [file cancers-15-00085-s001.zip › cancers-2110369-supplementary.pdf]

Supplementary material

Potent anticancer activity of CXCR4-targeted nanostructured toxins in aggressive endometrial cancer models

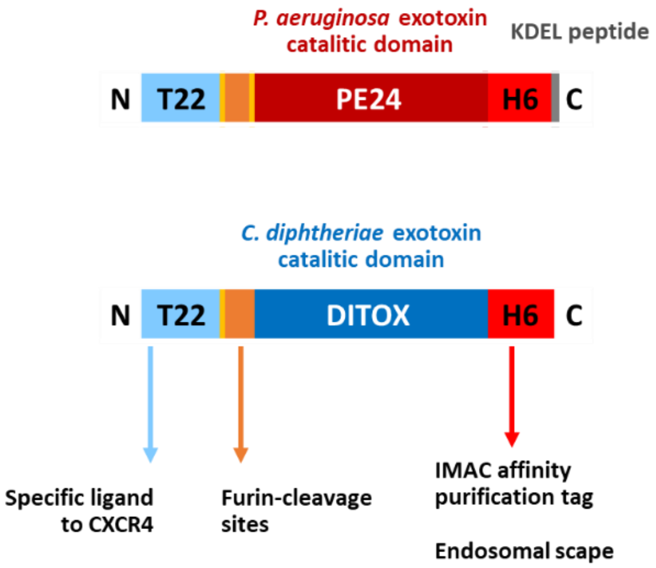

Figure S1. Schematic design of the CXCR4-targeted nanotoxins T22-PE24-H6 and T22-DITOX-H6. Adapted from [12].

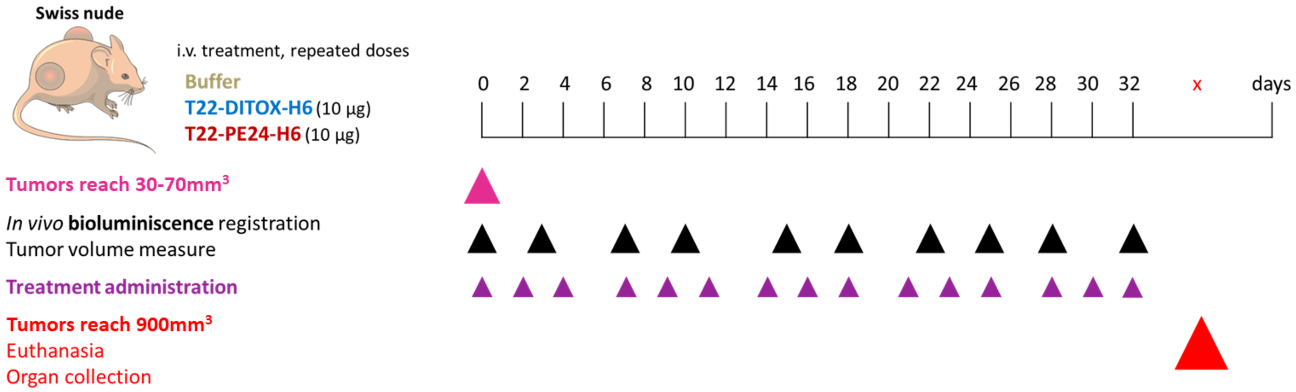

Figure S2. Experimental design of the in vivo assay used to assess the antitumor activity of CXCR4-targeted nanotoxins T22-PE24-H6 and T22-DITOX-H6 at repeated dosage (10 µg, q3w x15 doses) in the $CXCR4^{+}$ AN3CA subcutaneous EC model.

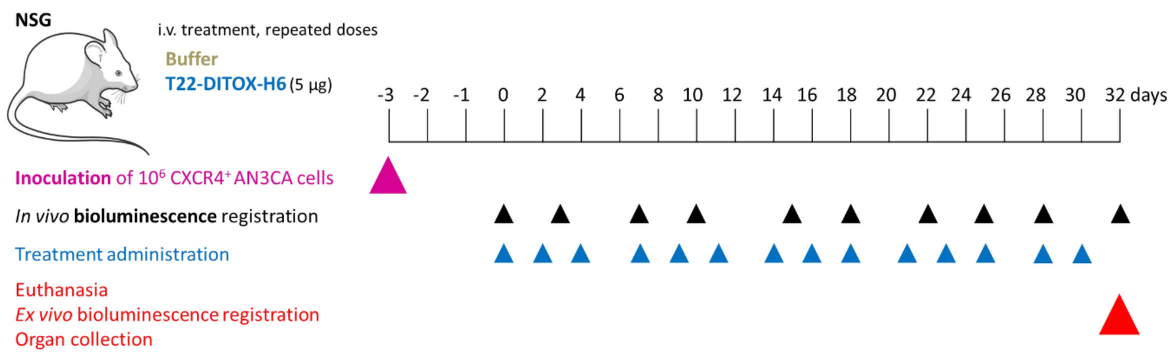

Figure S3. Experimental design of the in vivo assay used to assess the antimetastatic activity of CXCR4-targeted nanotoxin T22-DITOX-H6 at repeated dosage (5 µg, q3w x14 doses) in the $CXCR4^{+}$ AN3CA orthotopic EC model.

Table S1. Antimetastatic effect of repeated dosage of CXCR4-targeted nanoparticle T22-DITOX-H6 on metastases development in the $CXCR4^{+}$ AN3CA advanced endometrial cancer orthotopic model

	Liver metastasis					Lung mets.
	Total foci		Single cell foci	Cluster foci		Invaded tissue (%)
	Number	Area	Number	Number	Area	
**Control**	6,73 ± 1,54 [a]	1665,75 ± 1117,36 [b]	5,38 ± 1,27 [c]	2,36 ± 0,61 [d]	3424,54 ± 2487,89 [e]	8,34 ± 3,28 [f]
**T22-DITOX-H6**	1,05 ± 0,48 [a]	114,89 ± 21,50 [b]	1,03 ± 0,55 [c]	0,19 ± 0,07 [d]	199,56 ± 11,78 [e]	1,06 ± 1,09 [f]

Dissemination pattern to liver and lung observed in our xenograft orthotopic model of advanced endometrial cancer generated in NSG mice treated with a dosage of 5µg, 3q x 14 doses.
Results are reported as mean ± SEM of single cell number, number of cluster foci and their occupied area per mouse, in ten random sections with medium power microscope fields (200x magnification, 10 fields) in liver or lung sections, stained with human vimentin by immunohistochemistry. Comparison between groups was done using Mann-Whitney U statistical test. [a] p=0.002; [b] p=0.023; [c] p=0.005; [d] p=0.000; [e] p=0.001; [f] p=0.038
